# Real-world data of pyrotinib-based therapy for patients with brain metastases of HER2-positive advanced breast cancer: a single-center retrospective analysis and molecular portraits

**DOI:** 10.3389/fonc.2023.1105474

**Published:** 2023-06-16

**Authors:** Hui Wang, Qiaoyan Liu, Mi Zhang, Juan Zhang, Ran Ran, Yingying Ma, Jiao Yang, Fan Wang, Shujuan He, Xiaoai Zhao, Le Wang, Lingxiao Zhang, Danfeng Dong, Jin Yang

**Affiliations:** ^1^ Cancer Center, The First Affiliated Hospital of Xi’an Jiaotong University, Xi’an, China; ^2^ Department of Medical Oncology, The First Affiliated Hospital of Xi’an Jiaotong University, Xi’an, China; ^3^ Department of Oncology, Xi’an Ninth Hospital, Xi’an, China

**Keywords:** HER2-positive breast cancer, brain metastases, pyrotinib, clinical benefits, genomic profile

## Abstract

**Introduction:**

Pyrotinib is a novel irreversible pan-HER tyrosine kinase inhibitor (TKI). However, real-world data of pyrotinib-containing therapy in human epidermal growth factor receptor 2 (HER2)-positive metastatic breast cancer (MBC) and developing brain metastases (BMs) are limited, and the genomic profile of this subpopulation is almost undefined.

**Methods and materials:**

Patients with BM of HER2-positive MBC (n = 35) treated with pyrotinib-containing therapy were enrolled in this analysis. Progression-free survival (PFS), overall survival (OS), objective response rate (ORR), disease control rate (DCR), and toxicity profiles were evaluated. Hazard ratios (HRs) and 95% confidence intervals (CIs) for disease progression were estimated using the Cox proportional hazards models. Targeted next-generation sequencing of 618 cancer-relevant genes was performed on plasma and primary breast tumors from patients with BM and without BM.

**Results:**

The median PFS time was 8.00 (95% CI, 5.98–10.017) months, and the median OS time was 23 (95% CI, 10.412–35.588) months. The ORR was 45.7%, and the DCR was 74.3%. In the Cox multivariate analysis, prior exposure to brain radiotherapy (HR = 3.268), received pyrotinib as third- or higher-line treatment (HR = 4.949), subtentorial brain metastasis (HR = 6.222), and both supratentorial and subtentorial brain metastases (HR = 5.863) were independently associated with increased risk of progression. The frequent grade 3–4 adverse event was increased direct bilirubin (14.3%), and two patients suffered from grade 3–4 diarrhea. In the exploratory genomic analysis, altered frequencies of FGFR3, CD276, CDC73, and EPHX1 were higher in the BM group. The consistency of mutated profiles of plasma and primary lesion in the BM group was significantly lower (30.4% *vs.* 65.5%; *p* = 0.0038).

**Conclusions:**

Pyrotinib-containing therapy shows favorable effectiveness and tolerable safety in patients with BM of HER2-positive MBC, particularly in a population that is brain radiotherapy-naïve, received pyrotinib as first- or second-line treatment, and developed supratentorial brain metastasis. In the exploratory genomic analysis, patients with BM showed distinct genomic features from patients without BM.

## 1 Introduction

Human epidermal growth factor receptor 2 (HER2) amplification or overexpression presents in approximately 15%–20% of breast cancer and correlates with more aggressive clinical phenotype, increased metastatic potential, and shorter survival (1). Moreover, among breast cancer subtypes, HER2-positive breast cancers are predisposed to brain metastasis (BM) and are two to four times more likely to develop BM than those with HER2-negative tumors (2–4). According to the previous literature, BM has been reported in 10% to 16% of patients with early-stage HER2-positive breast cancers and 25% to 50% in the metastatic setting (5–7). As systemic therapies prolong the survival of patients with HER2-positive metastatic breast cancer (MBC), the incidence of developing BM is increasing (8, 9). While this subtype attains a great clinical benefit of effective HER2-targeted therapy, the prognosis of patients diagnosed with BM is generally poor (10, 11). In the subpopulation analysis of the registHER study, the median survival time after the first diagnosis of BM for total HER2-positive breast cancers was 13.0 months, and for patients diagnosed with BM subsequent to MBC diagnosis, it was 9.6 months (11). Therefore, there is an increasing need to optimize strategies targeting BM as well as to develop strategies for intracranial progression after initial therapy (12). Until recently, the optimal treatment strategy has been unsatisfactory, including the sequence or combination of whole brain radiotherapy (WBRT), stereotactic radiotherapy (SRT), stereotactic radiosurgery (SRS), and neurosurgery, systemic therapy, and intrathecal treatment methods (13). Although HER2 overexpression has been determined as a predictor of the response to anti-HER2 treatment, many drugs have a limited ability to cross the blood– brain barrier (BBB). Previous studies showed that trastuzumab did not decrease the incidence of developing BM despite responses achieved in extracerebral sites (9, 14). The management of novel systemic strategies to more effectively prevent or treat BM from HER2-positive MBC is needed, particularly in case local therapy is not possible or indicated (13, 15). Pyrotinib is a novel oral, second-generation, irreversible, pan-HER tyrosine kinase inhibitor (TKI) targeting HER1, HER2, and HER4, blocking the cell cycle in G1 phase and suppressing tumor growth (16, 17). Currently, pyrotinib has been widely exploited due to its small molecule property that enhances the ability to penetrate the BBB (18). Preclinical evidence and clinical data demonstrated the robust activity of pyrotinib inhibiting the proliferation of HER2-overexpressing breast tumors and the favorable efficacy of antitumor (16, 17, 19, 20). In the PHENIX and PHOEBE studies, pyrotinib showed favorable stability, good tolerance, and encouraging antitumor activity in patients with HER2-positive MBC previously treated with trastuzumab (21–23). Moreover, in a subgroup analysis of the PHENIX study, the pyrotinib plus capecitabine group had a longer progression-free survival (PFS) in patients with BM at baseline than the lapatinib plus capecitabine group (6.9 *vs.* 4.2 months; *p* = 0.011). In those without BM at baseline, fewer developed new BM in the pyrotinib group (23). In the phase II PERMEATE study, patients who had radiotherapy-naïve BM and experienced progressive disease after radiotherapy both achieved favorable intracranial objective response rates (74.6% and 42.1%, respectively). These results showed that pyrotinib-containing therapy was well tolerated and active for both intracranial and extracranial lesions in patients with BM of HER2-positive MBC (24). However, as many clinical trials in the metastatic setting explicitly excluded patients with BM, data from HER2-positive patients with BM are still lacking. It becomes an obstacle when aiming to investigate the optimal treatment for these patients, and more clinical research is needed.

Furthermore, progress is also hampered by the scarcity of collections of biological samples to attain annotated data and support translational research in this population (25). Studies have revealed that BM from breast cancer has a distinct genomic feature to that of the primary tumor, with the presence of mutations that are not detected in the primary tumor (25–27). However, biopsies of BM for genomic profile work are not readily available due to the high risks and complexities of neurosurgery and the fact that surgery may not always be appropriate in the presence of multiple lesions (28). Therefore, the usage of circulating tumor DNA (ctDNA) is increasing as a potentially useful further source of information on biology and response to therapy in patients with MBC and BM (29, 30). In a real-world study by Anwar et al., among eight of 168 patients with HER2-positive MBC and BM treated with pyrotinib, a high tumor mutational burden (TMB) was associated with poor survival (22). However, more information on the molecular profile of this subpopulation is needed.

This study aimed to determine the efficacy and safety profile of pyrotinib-based therapy in HER2-positive MBC and BM in the real world. Moreover, we used a sequencing assay to obtain the genomic features of this population.

## 2 Methods and materials

### 2.1 Patient population and data collection

From October 2018 to December 2021, 35 patients with HER2-positive MBC and BM at baseline treated with pyrotinib were enrolled in our real-world analysis. Eligible patients were women with histologically confirmed HER2-positive MBC and with a measurable lesion as defined by the revised Response Evaluation Criteria in Solid Tumors 1.1 (RECIST 1.1). Patients who discontinued pyrotinib treatment or developed BM during treatment of pyrotinib were excluded. Clinical information, including demographics and treatment history, was obtained through data tracking of the hospital’s medical records and telephone inquiries. All patients provided written informed consent for the use of their medical information for research purposes. The study involving human participants was reviewed and approved by the Ethics Committee of the First Affiliated Hospital of Xi’an Jiaotong University and conducted in accordance with the ethical guidelines of the Declaration of Helsinki.

### 2.2 Treatment and dose modification

Patients were prescribed pyrotinib-based therapy in routine clinical practice. The standard dosage was 400 mg orally once daily without breaks. The starting dose, dose adjustment, dose interruption, treatment discontinuation, and combined treatment with cytotoxic drugs and/or anti-HER2 drugs and/or brain radiotherapy were determined by physicians’ choice according to the results of previous clinical trials, the general health status, and wishes of patients. Information on clinical response was attained from imaging results, and imaging follow-up was scheduled every two drug cycles (21 days per cycle).

### 2.3 Sample collection and target next-generation sequencing

Seven of 35 patients with BM and another 10 patients without BM treated with pyrotinib underwent molecular profiling using a validated 618-gene panel next-generation sequencing (NGS) assay (**Figure S1**). All tumor tissues were pathologically reviewed to contain at least 20% tumor cells. Among the 17 patients enrolled, we collected 48 samples, including nine primary breast tumors and 39 peripheral blood samples (18 from patients with BM and 21 from patients without BM). We performed NGS of tumor and gDNA- matched germline DNA using the Accel-NGS 2S DNA Library Kit (Swift Biosciences, Inc., Ann Arbor, MI, USA) and the xGen Lockdown Probes kit (Integrated DNA Technologies, Inc., Coralville, IO, USA) for library preparation. The custom xGen Lockdown probe was synthesized by IDT for the exons and parts of introns of 618 genes. The prepared library was quantified using the Qubit 3.0 Fluorometer (Life Technologies, Inc., Carlsbad, CA, USA), and the quality and fragment size was measured using an Agilent 2100 Bioanalyzer (Agilent Technologies, Inc., Santa Clara, CA, USA). Samples underwent paired-end sequencing on an Illumina NextSeq CN500 platform (Illumina Inc., San Diego, CA, USA) with a 150-bp read length. The mean depth of coverage of 1,606× and 4,858× was achieved for tumor gDNA and blood ctDNA, respectively.

### 2.4 Data processing

Raw sequencing data were aligned to the reference human genome (the University of California Santa Cruz, genome on human —hg19, http://genome.ucsc.edu/index.html) by Burrows-Wheeler Aligner. After the removal of duplicates and local realignment, the Genome Analysis Toolkit was utilized for single- nucleotide variation, insertion, and deletion. Following the removal of the germline alterations from matched blood samples, the somatic alterations were then obtained. Variants were annotated using the ANNOVAR software tool. Copy number was analyzed using the CNVkit.

### 2.5 Statistical analysis

Descriptive analysis was utilized to document the demographic and clinical features of the patients. A comparison of clonal cluster numbers from seven patients with BM and nine of 10 patients without BM was performed using the *t-*test. Comparisons of TMB from seven patients with BM and 10 patients without BM and the discrepancy of consistency of mutated profiles of peripheral blood and primary lesion between patients with BM and without BM were performed using the Mann–Whitney *U*-test. The Kaplan–Meier plots for PFS and overall survival (OS) were performed for different factors, which were defined as the time from receiving pyrotinib treatment for BM to disease progression or death, and curves were compared using log-rank tests. The Cox multivariate proportional hazards model was also applied to identify risk factors of PFS. Variables with *p* < 0.2 in the univariate regression analysis and known to be significantly correlated with the prognosis of patients with brain metastases, even if they do not meet the set statistical screening criteria, were included in the Cox multivariate proportional hazards model. The mutated genes of patients with BM and without BM were executed by R (version 4.2.0, https://www.r-project.org/). The Kyoto Encyclopedia of Genes and Genomes (KEGG) pathways were conducted by the online Database for Annotation, Visualization and Integrated Discovery (DAVID; http://david.abcc.ncifcrf.gov/home.jsp). All statistical analyses were performed on SPSS version 25.0 (IBM SPSS, Armonk, NY, USA) or GraphPad Prism (version 8.3.0; GraphPad Software, La Jolla, CA, USA) software. Statistical significance was defined as a two-sided *p*- value <0.05.

## 3 Results

### 3.1 Baseline characteristics

A total of 35 breast cancer patients were recruited for this study, and it was necessary to clarify the clinical outcomes and characteristics from the time of brain metastasis and received pyrotinib treatment. Baseline characteristics were demonstrated in **Table 1**. The median age was 47 years with a range from 25 to 67 years. The majority of the patients (97.1%) had the invasive ductal carcinoma histological subtype. The percentages of grade II and grade III were 25.7% and 45.7%, respectively. Over half of the population (54.3%) were hormone receptor- positive, whereas 45.7% were hormone receptor- negative. A small proportion of patients (11.4%) harbored HER2 heterogeneity. Furthermore, patients often at T2 (68.8%), N1 (45.7%), and 20% were MBC at initial diagnosis. Extracranial metastases were observed in 88.6% of patients, the majority of which (57.1%) had lung metastases, 54.3% had bone metastases and followed by lymph nodes and/or soft tissue (45.7%) and liver metastases (40%), and the number of extracranial metastasis sites of most patients was more than two lesions. The number of BM of up to half of patients (48.6%) was ≥3. A large proportion of patients (40%) exhibited supratentorial and subtentorial metastases, followed by only supratentorial metastasis (31.4%) and only subtentorial metastasis (17.1%). Moreover, a small proportion of patients (n = 3) developed meningeal metastases. All of the patients had previously been treated with trastuzumab-containing treatment and other HER2-targeted therapies, including TKI-containing regimens (28.6%), pertuzumab-containing therapies (22.9%), and ado-trastuzumab emtansine (T-DM1) (2.9%).

**Table 1:** Clinical characteristics of HER2-positive breast cancers with brain metastasis.

Characteristics	No. (%) (n = 35)
Age	
Median (range), years	47 (25–67)
<50	19 (54.3)
≥50	16 (45.7)
The confirmed time, years	5.1 (1–15)
ECOG scale	
0–1	22 (62.9)
≥2	13 (37.1)
Primary tumor laterality	
Left	18 (51.4)
Right	16 (45.7)
Bilateral	1 (2.9)
Histology	
IDC	34 (97.1)
Other	1 (2.9)
Grade	
II	9 (25.7)
III	16 (45.7)
Unknown	10 (28.6)
HR status	
Positive	19 (54.3)
Negative	16 (45.7)
HER2 heterogeneity	
No	15 (42.9)
Yes	4 (11.4)
Unknown	16 (45.7)
T	
T1	4 (11.4)
T2	24 (68.6)
T3	2 (5.7)
T4	3 (8.6)
Tx	2 (5.7)
N	
N0	6 (17.1)
N1	16 (45.7)
N2	7 (20)
N3	6 (17.1)
Nx	
Stage at diagnosis	
I	3 (8.6)
II	12 (34.3)
III	11 (31.4)
IV	7 (20.0)
Unknown	2 (5.7)
No. of extracranial metastasis	
0	4 (11.4)
1	13 (37.1)
≥2	18 (51.4)
Extracranial metastasis sites	
Lymph nodes and/or soft tissue	16 (45.7)
Bone	19 (54.3)
Lung	20 (57.1)
Liver	14 (40.0)
No. of visceral metastases (including brain)	
1	10 (28.6)
2	15 (42.9)
≥3	10 (28.6)
No. of brain metastases	
<3	13 (37.1)
≥3	17 (48.6)
Unknown	5 (14.3)
Location of brain metastases	
Brain parenchyma metastases only	31 (88.6)
Subtentorial only	6 (17.1)
Supratentorial only	11 (31.4)
Supratentorial and subtentorial	12 (34.3)
Brain parenchyma and meningeal metastases	2 (5.7)
Supratentorial and subtentorial	2 (5.7)
Meningeal metastases only	1 (2.9)
Unknown	1 (2.9)
Symptoms of brain metastases	
Yes	14 (40.0)
No	18 (51.4)
Unknown	3 (8.6)
Resistance to prior trastuzumab	
Sensitive	6 (17.1)
Primary resistance	7 (20.0)
Acquired resistance	22 (62.9)
Prior brain radiotherapy	
**Yes**	13 (37.1)
**No**	22 (62.9)
Prior HER2-targeted therapy	
Trastuzumab	35 (100)
Pertuzumab	8 (22.9)
TKIs	10 (28.6)
T-DM1	1 (2.9)
Patient’s vital status	
Alive	22 (62.9)
Deceased	13 (37.1)
**Total**	35 (100.0)

Values are presented as number (%). Visceral metastases referred to lung, liver, brain, pleural, and peritoneal involvement. HER2 heterogeneity: the HER2 status of the primary and metastatic lesions was inconsistent.

ECOG, Eastern Cooperative Oncology Group; HER2, human epidermal growth factor receptor 2; HR, hormone receptor; IDC, infiltrating ductal carcinoma; T-DM1, ado-trastuzumab emtansine; TKIs, tyrosine kinase inhibitors.

### 3.2 Treatment administration

The information on treatment administration is shown in **Table 2**. All of the patients were exposed to pyrotinib in combination with chemotherapy. The most common chemotherapy regimens were capecitabine (51.4%), vinorelbine (17.1%), taxan (25.7%), and eribulin (2.9%). Moreover, a large proportion of patients (60%) received treatment with pyrotinib in combination with trastuzumab. Of 13 patients who were administrated with local therapy for BM, eight patients (22.9%) were treated with Gamma Knife, and five patients (14.3%) were treated with whole brain radiotherapy. Thirty-three patients (94.3%) received pyrotinib-containing treatment as the second-line or further lines of systematic therapy. Up to half of patients (48.6%) received pyrotinib-containing therapy across lines. Most enrolled patients (57.1%) received 400 mg of pyrotinib at baseline, and seven (20.0%) patients started with 320 mg/day, six (17.1%) patients started with 240 mg/day, and two (5.7%) patients had a starting dose of 160 mg/day. Nine (25.8%) patients experienced dose reduction.

**Table 2:** Treatment administration.

Pyrotinib treatment	No. (%) (n = 35)
Regimens	
Combined chemotherapy regimen	
Capecitabine	18 (51.4)
Vinorelbine	6 (17.1)
Taxane	9 (25.7)
Eribulin	1 (2.9)
Other	1 (2.9)
Combined with trastuzumab	
Yes	21 (60.0)
No	14 (40.0)
Combined with local therapy for brain metastasis	
Gamma Knife	8 (22.9)
Whole brain radiotherapy	5 (14.3)
Unknown	2 (5.7)
None	20 (57.1)
Lines of systematic therapy of pyrotinib	
1	2 (5.7)
2	11 (31.4)
≥3	22 (62.9)
Across lines therapy of pyrotinib	
Yes	17 (48.6)
No	18 (51.4)
Duration of pyrotinib (months)	
<3	3 (8.6)
≥3 to <12	13 (37.1)
≥12	19 (54.3)
Dosage Starting dosage (mg/day)	
400	20 (57.1)
320	7 (20.0)
240	6 (17.1)
160	2 (5.7)
Dosage reduction (mg/day)	
400→320	4 (11.4)
400→240	1 (2.9)
320→240	1 ((2.9)
240→160	3 (8.6)
**Total**	35 (100.0)

Values are presented as number (%). Across lines of therapy of pyrotinib: patients who received multiple lines of pyrotinib.

### 3.3 Treatment outcomes

**Figure 1:**
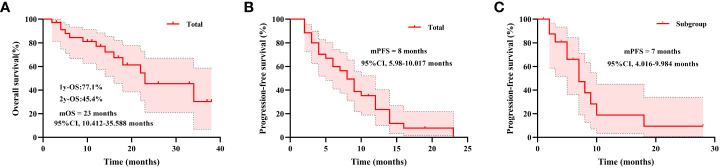
Kaplan–Meier estimates of overall survival for total patients **(A)**, progression-free survival for total patients **(B)**, and progression-free survival for patients who received across lines therapy of pyrotinib **(C)**. mPFS, median progression-free survival; mOS, median overall survival.

The median follow-up duration was 19 months. For total patients, the numbers of PFS and OS events were 28 (80.0%) and 13 (37.1%), respectively. The median OS time was 23 (95% CI, 10.412–35.588) months, and 1- year OS for total patients was 77.1% and 2-year OS was 45.4% (**Figure 1A**). The median PFS time was 8.00 (95% CI, 5.98–10.017) months (**Figure 1B**). In the subset of 17 patients who received across lines therapy of pyrotinib, the numbers of PFS events were 11 (64.7%). The median PFS time was 7.00 (95% CI, 4.016–9.984) months (**Figure 1C**).

Based on the results of univariate regression analysis (**Table S1**) and evidence of clinical expertise, the Cox multivariate proportional hazards model included the number of brain metastases (*p* = 0.0843), number of visceral metastases (*p* = 0.0361), previous brain radiotherapy (*p* = 0.0236), prior treatment by TKIs (*p* = 0.021), treatment lines for pyrotinib in the metastatic setting (*p* = 0.0604), lung metastasis (*p* = 0.1011), liver metastases (*p* = 0.229), location of brain metastases (*p* = 0.6226, **Figure S3A**) (31), combined with trastuzumab (*p* = 0.6868) (32), hormone receptor (*p* = 0.6633) (33), and prior exposure to endocrine therapy (*p* = 0.316) (34). In the Cox multivariate analysis, prior exposure to brain radiotherapy was independently associated with increased risk of progression (HR = 3.268; 95% CI, 1.018–10.493; *p* = 0.047; **Table 3**). M oreover, the log-rank test results indicated that for patients who did not receive brain radiotherapy previously, the median PFS was significantly longer (12 *vs.* 6 months; *p* = 0.0236; **Figure S2A**). Received pyrotinib as third-line or higher- line treatment was independently associated with increased risk of progression after treatment of pyrotinib for BM in the Cox multivariate analysis (HR = 4.949; 95% CI, 1.071–22.880; *p* = 0.041; **Table 3**), while received pyrotinib as first- or second-line treatment seemed to prolong PFS time (12 *vs.* 6 months), but the difference was not significant in log-rank test (*p* = 0.0604; **Figure S2D**). Moreover, in the Cox multivariate analysis, subtentorial brain metastasis (HR = 6.222; 95% CI, 1.264–30.638; *p* = 0.025) and both supratentorial and subtentorial brain metastases (HR = 5.863; 95% CI, 1.051–32.717; *p* = 0.044) were independently associated with increased risk of progression after treatment of pyrotinib for BM (**Table 3**). However, no significant difference in the median PFS was observed among patients with distinct locations of BM in the log-rank test analysis (**Figure S3A**). In the Cox multivariate analysis, prior exposure to TKIs and the number of visceral metastatic sites were not independently associated with increased risk of progression after treatment of pyrotinib for BM (**Table 3**). However, for patients who received TKIs previously, the median PFS was significantly shorter than that of patients who did not receive TKIs in the log-rank test (4.5 *vs.* 9 months; *p* = 0.021; **Figure S2B**). For patients who developed one to two visceral metastatic sites (including the brain), the median PFS was significantly longer than that of patients who developed ≥3 visceral metastatic sites (9 *vs.* 5 months; *p* = 0.0361; **Figure S2C**). Moreover, in the Cox multivariate analysis, the number of BM and received pyrotinib in combination with trastuzumab were not independently associated with increased risk of progression after treatment of pyrotinib for BM (**Table 3**). In a log-rank test, no significant difference in the median PFS was observed among patients with a distinct number of BM, with or without liver metastases, and who received pyrotinib in combination with trastuzumab (**Figure S3B-D**).

**Table 3:** Cox multivariate regression analyses of factors associated with PFS of patients.

Variables	HR (95% CI)	*p*- Value
Number of brain metastases (≥3 *vs.* 1–2)	1.405 (0.389–5.078)	0.604
Number of visceral metastases (including brain, ≥3 *vs.* 1–2)	6.255 (0.767–51.000)	0.087
Location of brain metastases (subtentorial *vs.* supratentorial)	6.222 (1.264–30.638)	0.025
Location of brain metastases (supratentorial and subtentorial *vs.* supratentorial)	5.863 (1.051–32.717)	0.044
Previous brain radiotherapy (yes *vs.* no)	3.268 (1.018–10.493)	0.047
Prior treatment by TKIs (yes *vs.* no)	1.316 (0.308–5.616)	0.711
Treatment lines for pyrotinib in metastatic setting (≥3 *vs.* 1–2)	4.949 (1.071–22.880)	0.041
Combined with trastuzumab (yes *vs.* no)	0.380 (0.129–1.117)	0.079

Model was adjusted by hormone receptors, prior exposure to endocrine therapy, lung metastasis, and liver metastases. Visceral metastases referred to lung, liver, brain, pleural, and peritoneal involvement.

CI, confidence interval; HR, hazard ratio; PFS, progression-free survival; TKIs, tyrosine kinase inhibitors.

All of the patients were included in the response analysis. The objective response rate (ORR) was 45.7%, and the disease control rate (DCR) was 74.3%, with two (5.7%) patients achieving complete response (CR), 24 (40%) patients achieving partial response (PR), and 10 (28.6%) patients achieving stable disease (SD). Nine (25.7%) patients had progressive disease (**Table 4**).

**Table 4:** The overall response rate and disease control rate of pyrotinib in HER2-positive breast cancers with brain metastasis.

Best response	No. (%) (n = 35)
CR	2 (5.7)
PR	14 (40.0)
SD	10 (28.6)
PD	9 (25.7)
ORR	14 (45.7)
DCR	24 (74.3)

Values are presented as number (%).

CR, complete response; DCR, disease control rate; HER2, human epidermal growth factor receptor 2; ORR, objective response rate; PD, progressive disease; PR, partial response; SD, stable disease.

### 3.4 Safety

As we used a patient self-reporting system to document adverse events (AEs) and given the retrospective nature of the study, omission in reporting AEs was unavoidable. Here, we reported the AEs of patients (**Table 5**). The most common AEs were diarrhea, anemia, increased direct bilirubin (DBIL), and hypoalbuminemia (all 65.7%). The most frequent grade 3–4 AE was increased DBIL (14.3%), followed by hypokalemia (11.5%), and only two patients (5.7%) suffered from grade 3–4 leukopenia, neutropenia, anemia, thrombocytopenia, and diarrhea. No treatment-related deaths were reported.

**Table 5:** Adverse events of patients.

Adverse event	All patients (n = 35), n (%)	Grade 1, n (%)	Grade 2, n (%)	Grade 3, n (%)	Grade 4, n (%)
Leukopenia	14 (40.0)	5 (14.3)	7 (20.0)	1 (2.9)	1 (2.9)
Neutropenia	10 (28.6)	5 (14.3)	3 (8.6)	–	2 (5.7)
Anemia	23 (65.7)	12 (34.3)	9 (25.7)	2 (5.7)	–
Thrombocytopenia	5 (14.3)	1 (2.9)	2 (5.7)	2 (5.7)	–
Hyperbilirubinemia	14 (40.0)	5 (14.3)	8 (22.9)	1 (2.9)	–
Increased DBIL	23 (65.7)	7 (20.0)	11 (31.4)	4 (11.4)	1 (2.9)
Increased IBIL	10 (28.6)	6 (17.1)	4 (11.4)	–	–
Hypoalbuminemia	23 (65.7)	17 (48.6)	6 (17.1)	–	–
Increased GPT	6 (17.1)	6 (17.1)	–	–	–
Increased GOT	10 (28.6)	9 (25.7)	1 (2.9)	–	–
Increased ALP	15 (42.9)	10 (28.6)	5 (14.3)	–	–
Increased creatinine	7 (20.0)	6 (17.1)	1 (2.9)	–	–
Hypokalemia	20 (57.1)	10 (28.6)	6 (17.1)	3 (8.6)	1 (2.9)
Hyponatremia	7 (20.0)	4 (11.4)	2 (5.7)	1 (2.9)	–
Hypocalcemia	20 (57.1)	8 (22.9)	11 (31.4)	1 (2.9)	–
Diarrhea	23 (65.7)	9 (25.7)	12 (34.3)	2 (5.7)	–
Nausea/vomit	14 (40.0)	9 (25.7)	3 (8.6)	2 (5.7)	–
Hand-foot syndrome	9 (25.7)	4 (11.4)	4 (11.4)	1 (2.9)	–
Oral mucositis	3 (8.6)	2 (5.7)	1 (2.9)	–	–
Anorexia	8 (22.9)	7 (20.0)	1 (2.9)	–	–
Asthenia	5 (14.3)	5 (14.3)	–	–	–
Rash	4 (11.4)	3 (8.6)	1 (2.9)	–	–
Abdominal discomfort	2 (5.7)	1 (2.9)	1 (2.9)	–	–
Urinary tract infection	2 (5.7)	2 (5.7)	–	–	–

Values are presented as number (%).

ALP, alkaline phosphatase; DBIL, direct bilirubin; GOT, glutamic oxaloacetic transaminase; GPT, glutamic pyruvic transaminase; IBIL, indirect bilirubin.

### 3.5 Distinct mutational profile of patients with BM and without BM and pathway enrichment analyses of patients with BM

**Figure 2:**
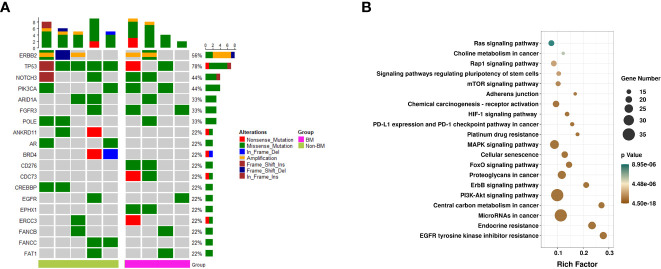
Mutational landscape and KEGG pathways. **(A)** Mutational landscape of primary breast tumors from four of seven patients with BM and five of 10 patients without BM. Displayed are mutations, indels, and amplification likely to be pathogenic, summarized by gene for each patient. Bottom bar refers to group (BM and non-BM). Right, variant classification of the alterations within each gene. **(B)** KEGG pathways of all samples from patients with BM (including 17 plasma samples and four tissue samples of primary breast tumors). The y-axis represents KEGG-enriched terms. The x-axis represents the rich factor. The size of the dot represents the number of genes under a specific term. The color of the dots represents the adjusted *p*-value. BM, brain metastasis; KEGG, Kyoto Encyclopedia of Genes and Genomes.

Previous literature showed that patients who developed BM harbored distinct genomic features (25, 35). We next identified the mutational profile of primary breast tumors from patients with BM and without BM through the target next-generation sequencing of 618 genes (**Table S2**). Ultimately, samples of primary breast tumors were collected from nine patients with HER2-positive MBC, including four of seven patients with BM and five of 10 patients without BM. The clinicopathological characteristics of patients and samples are shown in **Table S3**. Overall, the most frequently mutated genes (**Figure 2A**) in total samples were *TP53* (7/9, 78%). The mutation of *FGFR3* was found more frequently in the BM group than in the non-BM group (2/4 *vs.* 1/5), and two patients in the BM group presented mutations of *CD276*, *CDC73*, and *EPHX1*, while mutations of these genes were not detected in the non-BM group. The altered frequency of *TP53* was higher in the non-BM group than in the BM group (5/5 *vs.* 2/4, respectively). Two patients in the non-BM group presented mutations of *ANKRD11*, *AR*, *BRD4*, *CREBBP*, and *FANCC*, while mutations of these genes were not detected in the BM group. The profile of genetic alterations was furtherly investigated in samples of brain metastases from HER2-positive breast cancer. Data were obtained from an open-access cancer genomics database, Memorial Sloan Kettering Cancer Center (MSKCC), by using the cBioPortal for Cancer Genomics (http://cbioportal.org). The details of the mutated genes of MSKCC are shown in **Table S4**. We found that *TP53* was also the most frequently altered gene in this cohort, which was higher than that in our BM group (9/12 *vs.* 2/4). In addition, mutations of *CD276* and *PIK3CA* were also found in this database. We furtherly conducted the pathway enrichment analyses to determine the functional characteristics of the mutational genes of 21 samples from patients with BM (including 17 plasma samples and four tissue samples of primary breast tumors) (**Figure S1**). The results revealed the genes were significantly over-represented in central carbon metabolism, PI3K-AKT, ErbB, and Foxo signaling pathways (**Figure 2B**).

### 3.6 Discrepancies of TMB, number of clusters, and consistency of ctDNA and primary lesion in patients with BM and without BM

**Figure 3:**
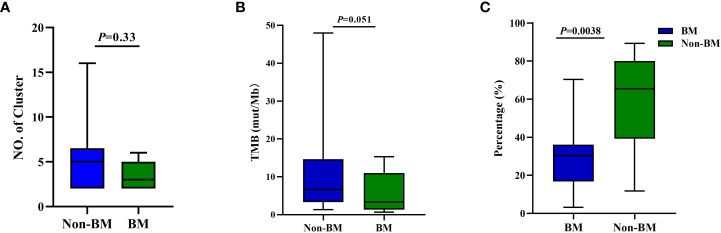
Discrepancies of TMB, number of clusters, and consistency of mutated profiles of peripheral blood and primary lesion in patients with brain metastasis and without brain metastasis. **(A)** Clonal cluster number from seven patients with BM and nine of 10 patients without BM; *p*- values by *t*-test. **(B)** TMB from seven patients with BM and 10 patients without BM; *p* -values by Mann–Whitney *U*-test. **(C)** Discrepancy of consistency of mutated profiles of peripheral blood and primary lesion between patients with BM and without BM; *p* -values by Mann–Whitney *U*-test. BM, brain metastasis; TMB, tumor mutational burden.

We furtherly investigated the discrepancies in other genomic features between patients with BM and without BM. We found that there was no difference in the number of clonal clusters based on primary tissue and serial ctDNA (**Figure 3A**) and TMB between patients with BM and without BM (*p* = 0.33 and *p* = 0.051, respectively; **Figure 3B**). However, the consistency of mutated profiles of peripheral blood and primary lesion in the non-BM group was significantly higher than that in the BM group (65.5% *vs.* 30.4%; *p* = 0.0038; **Figure 3C**).

## 4 Discussion

Patients with HER2-positive breast cancer harbor aggressive biological behavior and with a high risk of BM (2, 3). The clinical benefits conferred by HER2-targeted therapy allow patients to live longer; therefore, there is enough time to develop BM (9). Generally, systematic therapy is the standard of care for BM if there is progressive disease following local therapy, while the efficacy has been unsatisfactory. Pyrotinib is a novel oral, second-generation, irreversible, pan-HER TKI. Results from the phase 3 PHOEBE (36) and PHENIX (23) studies have shown the favorable effects of pyrotinib in patients with HER2-positive MBC after trastuzumab. The PERMEATE trial (24), subpopulation analysis of the PHENIX trail (23), and retrospective real-world studies (22, 37) showed the activity with pyrotinib against BM. Our study investigated the real-world data of pyrotinib in patients with BM at baseline in complicated clinical practice and supplemented the findings of clinical trials.

Our study showed that patients with BM at baseline obtained distinctly clinical benefits with a median PFS of 8.00 months and yielded a favorable median PFS of 7 months in the subgroup who received therapy of pyrotinib across lines. It was similar to the median PFS of 8.00 months in the real-world study (n = 168) by Anwar et al. (22). However, the subpopulation with BM at baseline in the PHENIX trial yielded a median PFS of 6.9 months (23). We guessed the prolonged PFS in our study partly attributed to the treatment of pyrotinib in combination with trastuzumab (60%). The effective antitumor activity and well-tolerated safety of dual HER2 blockade with TKI plus trastuzumab have been reported previously (38). Moreover, a single-arm exploratory phase II trial was designed to assess the efficacy and safety of pyrotinib plus trastuzumab and chemotherapy in patients with HER2-positive MBC. The median PFS was 9.4 months in patients with BM (95% CI, 6.6–12.1 months) (39). However, further verification is needed in the future due to the small sample size of our study and its nature. These findings revealed that the treatment of pyrotinib-containing brought promising clinical benefits and powerful clinical efficacy across lines of therapy for patients with HER2-positive MBC and BM. In the phase 2 PERMEATE study, patients who had radiotherapy-naïve BM (cohort A) yielded a higher central nervous system (CNS) ORR and longer median PFS than those who had progressive disease of BM after radiotherapy (cohort B) (74.6% *vs.* 46%; 11.3 *vs.* 5.6 months, respectively) (24). In a phase II trial, treatment of lapatinib for BM also had low response rates after brain radiotherapy (40, 41). Similarly, in our work, we found that prior exposure to brain radiotherapy was an independent factor for poor PFS (no prior exposure *vs.* prior exposure: HR = 0.150; 95% CI, 0.023–0.971; *p* = 0.046). Taken together, these results indicated that the clinical response of TKI-containing therapy was poor after brain radiotherapy compared to those who are brain radiotherapy-naïve (18). Moreover, pyrotinib-based therapy was generally well-tolerated (23, 24). The frequent grade 3– 4 AEs were DBIL, hypokalemia, and diarrhea, which were consistent with reports of the previous clinical trial (23) and obviously increased DBIL partly owing to developing liver metastases. No severe AE was reported.

Owing to small-molecular mass, other TKIs have also been investigated as a promising treatment strategy to treat BM (22). Lapatinib was a small dual tyrosine-kinase inhibitor of HER1 and HER2. A phase III trial of capecitabine with or without lapatinib suggested that lapatinib may reduce the risk of disease progression to the CNS (42). In EMILIA (43) and LANDSCAPE (44) trials, among patients with BM at baseline or with new brain lesions, the median PFS was 5.7 and 5.5 months in the lapatinib plus capecitabine arm. In the phase 2 TBCRC 022 study, neratinib was effective against refractory, HER2-positive breast cancer BM, with CNS ORR of 49% in the lapatinib-naïve cohort and 33% in the lapatinib-pretreated cohort (45). In the HER2CLIMB trial, patients were divided into trastuzumab plus capecitabine with tucatinib, or placebo arms, and up to half of the participants had BM at baseline. In the subset of 291 patients with measurable BM, the risk of progression in the brain or death was reduced by 68% in the triple combination arm (HR = 0.32; 95% CI, 0.22– 0.48; *p* = 0.0001). The median duration of CNS PFS was 9.9 months (95% CI, 8.0– 13.9 months) in the triple combination arm. Moreover, among the 174 patients with active BMs, the risk of progression in the brain or death was reduced by 64% in the triple combination arm (HR = 0.36; 95% CI, 0.22– 0.57; *p* = 0.0001). The median duration of CNS PFS was 9.5 months (95% CI, 7.5– 11.1 months) in the triple combination arm (46, 47). The favorable results of this study provided direct trial evidence; thus, the American Society of Clinical Oncology (ASCO), the Society for Neuro-Oncology (SNO), and the American Society for Radiation Oncology (ASTRO) recommended that the combination of tucatinib, trastuzumab, and capecitabine may be used as the preferred systemic treatment for patients with HER2-positive MBC and active BM and with the progressed disease after receiving trastuzumab, pertuzumab, and/or T-DM1 (48). Taken together, these results proved that TKI-containing therapy had promising activity in patients with HER2-positive MBC and BM, even those with active BM.

With the development of anti-HER2 drugs, patients with BM have more treatment options. However, some HER2-targeted drugs showed limited concentration ability in the brain, especially those with large molecular weight, like trastuzumab and T-DM1 (30, 49). In a literature-based meta-analysis of three large-scale, phase III, adjuvant trastuzumab trials (N = 6,738) conducted by Bria et al., they found the incidence of BM was significantly higher in the trastuzumab-containing treatment arms compared with the non-trastuzumab-containing arms (absolute difference, 0.62%), and the relative risk of BM was 1.57 (95% CI, 1.03– 2.37) (50). It was proved by Stemmler et al. that trastuzumab was able to cross the BBB when the BBB was impaired (51), and, after WBRT, the radiologic responses (74.2%) and the median interval to brain progression (10.5 months) were promising in patients treated with trastuzumab (52). In a phase II trial (PATRICIA), patients with HER2-positive MBC and BM received pertuzumab in combination with high-dose trastuzumab (6 mg/kg weekly) and obtained the clinical benefit rate (CBR) at 4 and 6 months of 68% and 51%, respectively (7). These results revealed that trastuzumab showed limited efficacy in patients with BM, unless trastuzumab is administrated after WBRT, although this needs to be confirmed by a large-scale trial. In preclinical work, the administration of trastuzumab by intracerebral micro-infusion significantly prolonged OS compared to the rats administrated with trastuzumab systemically (53). A clinical case reported the effect of introducing trastuzumab into the cerebrospinal fluid of patients *via* intrathecal administration for the treatment of BM (54). It is important to note, however, that there is no intrathecal formulation of trastuzumab available at present and that there are no safety data to support it (9). Median PFS with T-DM1 in patients with HER2-positive MBC and stable BM was similar between EMILIA (5.9 months) (43) and KAMILLA (5.5 months) (55). However, the median PFS among all randomized participants was 9.6 months (56). Trastuzumab deruxtecan (T-DXd and DS-8201) is a novel antibody–drug conjugate consisting of a humanized HER2-directed monoclonal antibody linked to a topoisomerase I inhibitor payload through a tetrapeptide-based cleavable linker and with high inhibitory potency and high membrane permeability (57). In the subgroup analysis from the randomized phase 3 study DESTINY-Breast03 and DESTINY-Breast01, the efficacy of trastuzumab deruxtecan was comparable in patients with BM at baseline (58, 59). Recently, the phase 2 TUXEDO-1 trial reported data regarding the potential activity of trastuzumab deruxtecan in active BM. The median PFS was 14 months, and the intracranial response rate was 73.3% in the intention-to-treat population and 78.6% in the per-protocol population (13). These results were provided as proof of principle for the intracranial activity of ADCs.

The age of genomic medicine has enabled us to identify the molecular features of tumors, facilitate the diagnosis and assessment of disease progression, and support translational research (25). In several exploratory studies, patients who developed BM presented frequently genes altered in HER2 or EGFR/PTEN drive pathway in primary BC (27, 60), had a higher positivity of immunotherapy biomarkers (including TMB, MSI, and CD274) (61), and had more copy-number alterations and more actionable genetic alterations in BM than in primary BC (35). Similarly, we also found that the consistency of mutated profiles of peripheral blood and primary lesion in the BM group was lower than that in the non-BM group (65.5% *vs.* 30.4%; *p* = 0.0038). We found that patients who developed BM had more frequent mutations of *PIK3CA*, *FGFR3*, *CD276*, *CDC73*, and *EPHX*. Among these genes, B7-H3 (CD276), as a member of the B7/CD28 superfamily, is an important factor in downregulating immune responses against tumors. Its overexpression correlated with invasive metastatic potential and poor prognosis in pediatric brain tumors (62) and had a negative relation with vascular endothelial growth factor expression (63). Because of tumor selectivity in the brain and the actionability of targeted therapeutic agents to B7-H3, therapeutics directed to this antigen have potential utility in various CNS tumors. These findings were favorable to identify the subsets of BC for developing BM and to define subgroups of patients who potentially could benefit from novel targeted therapies.

However, some limitations should be acknowledged. Primary limitations were inherent to the observational retrospective study design. These included missing information about some baseline characteristics and incomplete documentation about treatment toxicities. Additionally, the follow-up period is relatively short, and further analysis of the data will be needed after a longer period of follow-up. Moreover, we mainly focused on the efficacy of pyrotinib-based therapy in patients with brain metastasis, and there was a lack of real-world data regarding tucatinib and T-DXd, which were the current standards of care in brain metastasis in breast cancer. Finally, the underpowered nature of the sequencing analyses resulted in limited knowledge of the genomic profile of patients developing BM.

## 5 Conclusions

This study indicated the favorable efficacy and tolerable safety of pyrotinib-containing therapy in patients with HER2-positive MBC and BM, particularly in a population with brain radiotherapy-naïve, received pyrotinib as first- or second-line treatment, and developed supratentorial brain metastasis. In the exploratory genomic analysis, we identified the genomic features of patients with BM of HER2- positive MBC. However, more systematic studies are needed and should include a larger sample size to identify molecular features in patients with BM and extend the novel therapeutic options.

## Data availability statement

The datasets presented in this study can be found in online repositories. The names of the repository/repositories and accession number(s) can be found in the article/**Supplementary Material**.

## Ethics statement

The studies involving human participants were reviewed and approved by the Ethics Committee of the First Affiliated Hospital of Xi’an Jiaotong University. The patients/participants provided their written informed consent to participate in this study.

## Author contributions

Study concepts and design: HW, JinY, and DD. Performed the experiments: HW and QL. Acquisition of data, analysis, and interpretation of data: HW, QL, MZ, RR, YM, XZ, and FW. Data curation and resources: JiaY, JZ, LZ, LW, and SH. Manuscript preparation: HW, QL, JinY, and DD. Manuscript editing: all authors. Manuscript review: all authors. All authors contributed to the article and approved the submitted version.

## Supplementary material

Supplementary Figure 1 Schematic of genomic study flow.

Supplementary Figure 2 The analysis of progression−free survival in specified subgroups. **(A)** Kaplan-Meier plot of progression-free survival for patients with and without prior brain radiotherapy. **(B)** Kaplan-Meier plot of progression-free survival for patients with and without prior TKIs exposure; **(C)** Kaplan-Meier plot of progression-free survival for patients with 1-2 visceral metastatic sites and with ≥3 visceral metastatic sites. **(D)** Kaplan-Meier plot of progression-free survival for patients who received pyrotinib as first or second-line treatment and for patients who received pyrotinib as third or higher line treatment.CI, confidence interval; HR, hazard ratio; TKIs, tyrosine kinase inhibitors.

Supplementary Figure 3 The analysis of progression−free survival in specified subgroups. **(A)** Kaplan-Meier plot of progression-free survival for patients with distinct locations of brain metastases; **(B)** Kaplan-Meier plot of progression-free survival for patients with 1-2 brain metastatic sites and with ≥3 brain metastatic sites; **(C)** Kaplan-Meier plot of progression-free survival for patients received treatment of pyrotinib in combination with trastuzumab and without trastuzumab; **(D)** Kaplan-Meier plot of progression-free survival for patients with liver metastases and without liver metastases.
