# Supplementary figures and images for: Real-world data of pyrotinib-based therapy for patients with brain metastases of HER2-positive advanced breast cancer: a single-center retrospective analysis and molecular portraits
Source: Front Oncol. 2023 Jun 16;13:1105474. doi: 10.3389/fonc.2023.1105474 (PMC10313114; doi:10.3389/fonc.2023.1105474)

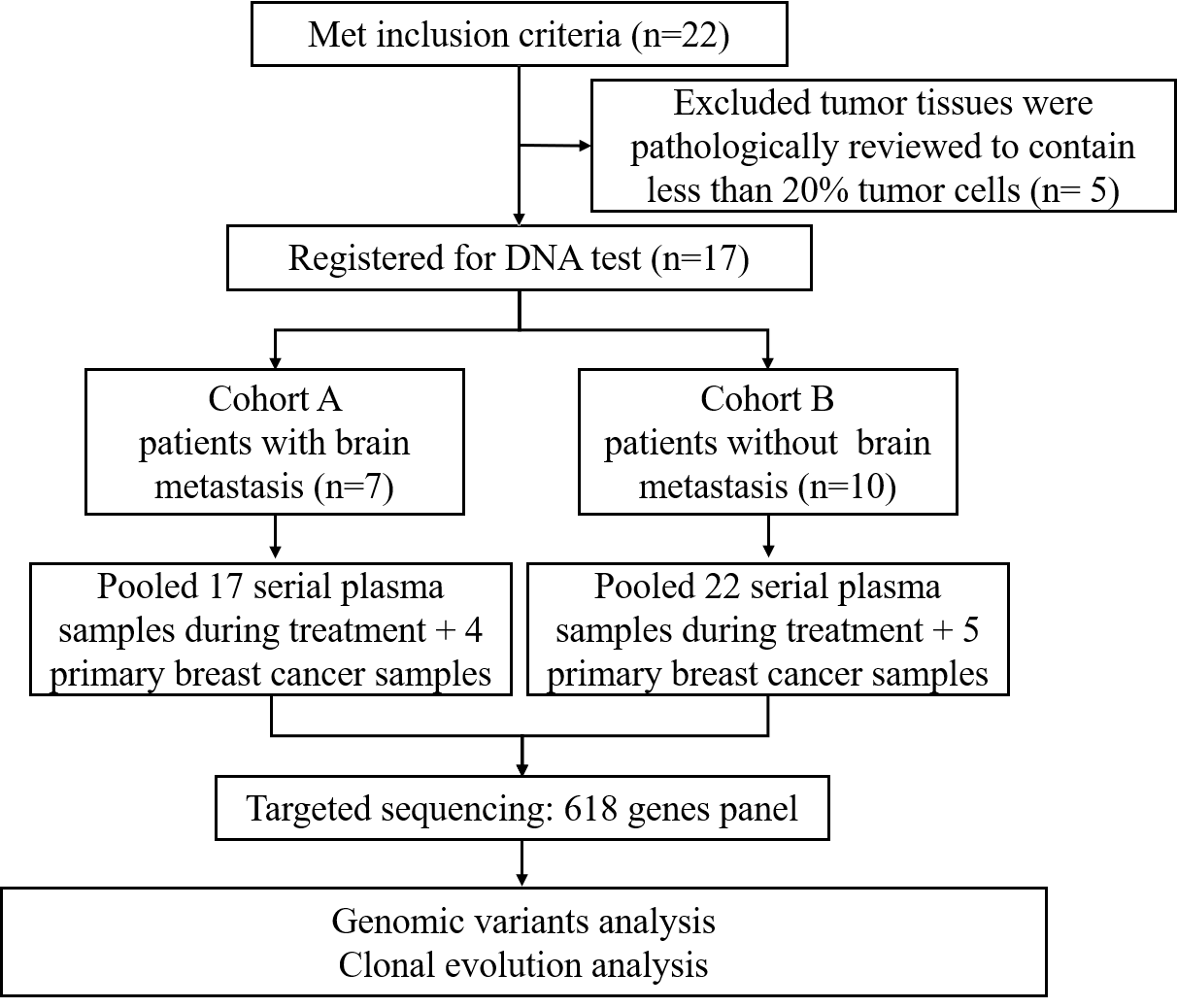

Supplement: Supplementary Figure 1 — Schematic of genomic study flow. [file Image_1.tif]

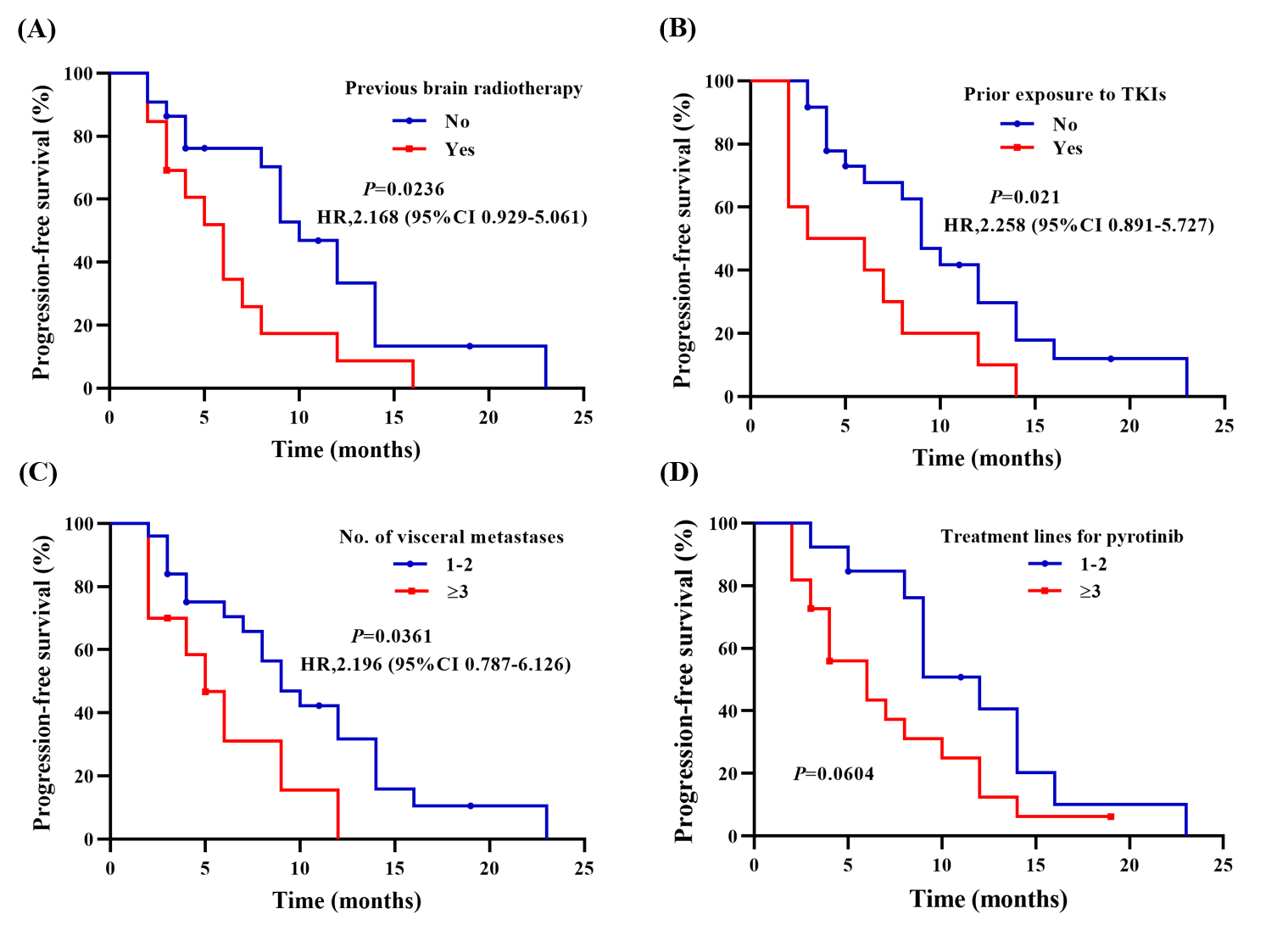

Supplement: Supplementary Figure 2 — The analysis of progression−free survival in specified subgroups. (A) Kaplan-Meier plot of progression-free survival for patients with and without prior brain radiotherapy. (B) Kaplan-Meier plot of progression-free survival for patients with and without prior TKIs exposure; (C) Kaplan-Meier plot of progression-free survival for patients with 1-2 visceral metastatic sites and with ≥3 visceral metastatic sites. (D) Kaplan-Meier plot of progression-free survival for patients who received pyrotinib as first or second-line treatment and for patients who received pyrotinib as third or higher line treatment.CI, confidence interval; HR, hazard ratio; TKIs, tyrosine kinase inhibitors. [file Image_2.tif]

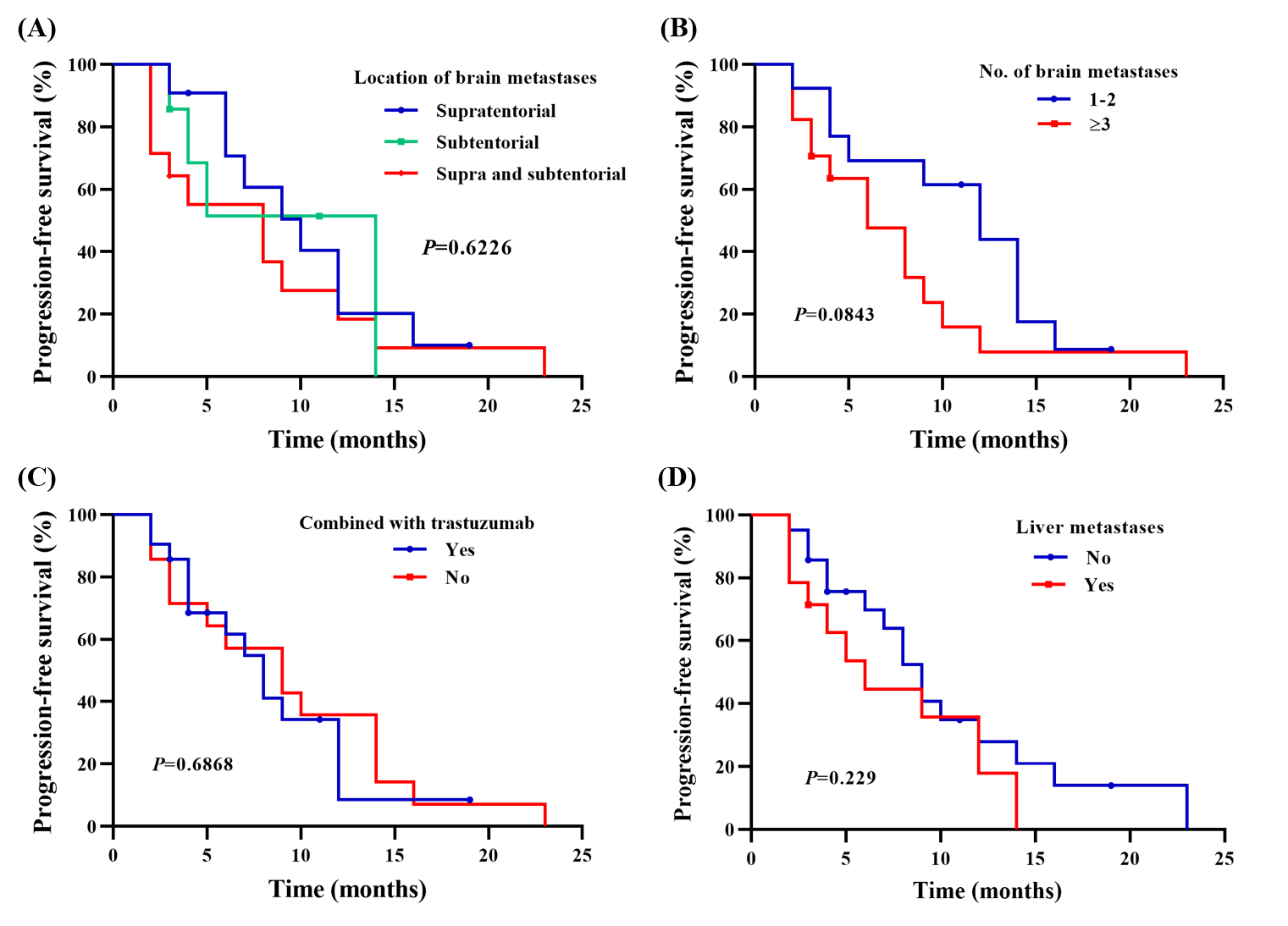

Supplement: Supplementary Figure 3 — The analysis of progression−free survival in specified subgroups. (A) Kaplan-Meier plot of progression-free survival for patients with distinct locations of brain metastases; (B) Kaplan-Meier plot of progression-free survival for patients with 1-2 brain metastatic sites and with ≥3 brain metastatic sites; (C) Kaplan-Meier plot of progression-free survival for patients received treatment of pyrotinib in combination with trastuzumab and without trastuzumab; (D) Kaplan-Meier plot of progression-free survival for patients with liver metastases and without liver metastases. [file Image_3.tif]
