# Supplementary material for: Real-world data of pyrotinib-based therapy for patients with brain metastases of HER2-positive advanced breast cancer: a single-center retrospective analysis and molecular portraits
Source: Front Oncol. 2023 Jun 16;13:1105474. doi: 10.3389/fonc.2023.1105474 (PMC10313114; doi:10.3389/fonc.2023.1105474)
Supplement: Supplementary file 4 [file Table_1.docx]

**Table S1.** **Univariate regression analysis of factors associated with PFS of patients.**

| **Variables** | ***P* value** | |
| --- | --- | --- |
| Hormone receptor status (positive vs negative) | 0.6633 |  |
| HER-2 heterogeneity (yes vs no) | 0.4818 |  |
| Stage at diagnosis (I-III vs IV) | 0.7890 |  |
| No. of extracranial metastasis (0-1 vs ≥2) | 0.3341 |  |
| Liver metastases (yes vs no) | **0.229** |  |
| Lung metastases (yes vs no) | **0.1011** |  |
| Number of visceral metastases (including brain, ≥3 *vs.* 1-2) | **0.0361** |  |
| Number of brain metastases (≥3 *vs.* 1-2) | **0.0843** |  |
| Location of brain metastases (subtentorial *vs.* supratentorial) | 0.8003 |  |
| Location of brain metastases (supra and subtentorial *vs.* supratentorial) | 0.3776 |  |
| Previous brain radiotherapy (yes *vs.* no) | **0.0236** |  |
| Prior treated by TKIs (yes *vs.* no) | **0.021** |  |
| Prior exposure to endocrine therapy (yes *vs.* no) | **0.316** |  |
| Treatment lines for pyrotinib in metastatic setting (≥3 *vs.* 1-2) | **0.0604** |  |
| Combined with trastuzumab (yes *vs.* no) | 0.6868 |  |

The bold factors were the variable that with P<0.2 in Univariate regression analysis and were included in the multivariate Cox proportional hazard model.
