# Supplementary material for: Real-world data of pyrotinib-based therapy for patients with brain metastases of HER2-positive advanced breast cancer: a single-center retrospective analysis and molecular portraits
Source: Front Oncol. 2023 Jun 16;13:1105474. doi: 10.3389/fonc.2023.1105474 (PMC10313114; doi:10.3389/fonc.2023.1105474)
Supplement: Supplementary file 5 [file Table_2.docx]

Table S2: List of target region of 618 cancer-related genes.

| **Gene** | **Gene** | **Gene** | **Gene** | **Gene** | **Gene** |
| --- | --- | --- | --- | --- | --- |
| ABL1 | ABL2 | ACVR1 | ACVR1B | ADGRA2 | AGO2 |
| AIP | AKT1 | AKT2 | AKT3 | ALK | ALOX12B |
| AMER1 | ANKRD11 | APC | AR | ARAF | ARFRP1 |
| ARID1A | ARID1B | ARID2 | ARID5B | ASXL1 | ASXL2 |
| ATM | ATR | ATRX | AURKA | AURKB | AXIN1 |
| AXIN2 | AXL | B2M | BABAM1 | BAP1 | BARD1 |
| BAX | BBC3 | BCL10 | BCL11A | BCL2 | BCL2L1 |
| BCL2L11 | BCL2L2 | BCL6 | BCOR | BCORL1 | BIRC3 |
| BIRC5 | BLCAP | BLK | BLM | BMPR1A | BRAF |
| BRCA1 | BRCA2 | BRD3 | BRD4 | BRIP1 | BTG1 |
| BTK | BUB1 | BUB1B | BUB3 | CALR | CARD11 |
| CARM1 | CASP8 | CBFB | CBL | CCND1 | CCND2 |
| CCND3 | CCNE1 | CD274 | CD276 | CD74 | CD79A |
| CD79B | CDC25C | CDC42 | CDC73 | CDH1 | CDK1 |
| CDK12 | CDK2 | CDK4 | CDK5 | CDK6 | CDK7 |
| CDK8 | CDK9 | CDKN1A | CDKN1B | CDKN1C | CDKN2A |
| CDKN2B | CDKN2C | CEBPA | CENPA | CHD1 | CHD2 |
| CHD3 | CHD4 | CHEK1 | CHEK2 | CIC | CREBBP |
| CRLF2 | CSDE1 | CSF1R | CSF3R | CTCF | CTLA4 |
| CTNNA1 | CTNNB1 | CUL3 | CXCL8 | CXCR4 | CYLD |
| CYSLTR2 | DAXX | DCUN1D1 | DDB2 | DDR2 | DICER1 |
| DIRAS3 | DIS3 | DIS3L2 | DNAJB1 | DNMT1 | DNMT3A |
| DNMT3B | DOT1L | DROSHA | DUSP4 | E2F1 | E2F3 |
| EED | EGF | EGFL7 | EGFR | EIF1AX | EIF4A2 |
| EIF4E | ELF3 | EMSY | EP300 | EPAS1 | EPCAM |
| EPHA3 | EPHA5 | EPHA7 | EPHB1 | ERBB2 | ERBB3 |
| ERBB4 | ERCC1 | ERCC2 | ERCC3 | ERCC4 | ERCC5 |
| ERF | ERG | ERRFI1 | ESR1 | ETV1 | ETV4 |
| ETV5 | ETV6 | EWSR1 | EXT1 | EXT2 | EZH1 |
| EZH2 | FAM175A | FAM46C | FAM58A | FANCA | FANCB |
| FANCC | FANCD2 | FANCE | FANCF | FANCG | FANCI |
| FANCL | FANCM | FAS | FAT1 | FAT4 | FBXW7 |
| FGF10 | FGF14 | FGF19 | FGF23 | FGF3 | FGF4 |
| FGF6 | FGFR1 | FGFR2 | FGFR3 | FGFR4 | FH |
| FLCN | FLT1 | FLT3 | FLT4 | FOLR3 | FOXA1 |
| FOXA2 | FOXL2 | FOXO1 | FOXP1 | FRS2 | FUBP1 |
| FYN | GABRA6 | GALNT12 | GATA1 | GATA2 | GATA3 |
| GATA4 | GATA6 | GEN1 | GID4 | GLI1 | GLI2 |
| GNA11 | GNA13 | GNAQ | GNAS | GOPC | GPC3 |
| GPS2 | GRB2 | GREM1 | GRIN2A | GRM3 | GSK3B |
| H3F3A | H3F3B | H3F3C | HDAC1 | HDAC2 | HDAC3 |
| HDAC4 | HDAC6 | HDAC8 | HGF | HIF1A | HIST1H1C |
| HIST1H2BD | HIST1H3A | HIST1H3B | HIST1H3C | HIST1H3D | HIST1H3E |
| HIST1H3F | HIST1H3G | HIST1H3H | HIST1H3I | HIST1H3J | HIST2H3C |
| HIST2H3D | HIST3H3 | HLA-A | HLA-B | HNF1A | HOXB13 |
| HRAS | HSD3B1 | ICOSLG | ID3 | IDH1 | IDH2 |
| IFNGR1 | IGF1 | IGF1R | IGF2 | IGF2R | IKBKE |
| IKZF1 | IL10 | IL7R | INHA | INHBA | INPP4A |
| INPP4B | INPPL1 | INSR | IRF2 | IRF4 | IRS1 |
| IRS2 | JAK1 | JAK2 | JAK3 | JUN | KAT6A |
| KDM5A | KDM5C | KDM6A | KDR | KEAP1 | KEL |
| KIT | KLF4 | KLHL6 | KMT2A | KMT2B | KMT2C |
| KMT2D | KMT5A | KNSTRN | KRAS | LATS1 | LATS2 |
| LMO1 | LRP1B | LRRK2 | LYN | LZTR1 | MAGI2 |
| MALT1 | MAP2K1 | MAP2K2 | MAP2K4 | MAP3K1 | MAP3K13 |
| MAP3K14 | MAP4K1 | MAPK1 | MAPK3 | MAPKAP1 | MAX |
| MCL1 | MDC1 | MDH2 | MDM2 | MDM4 | MED12 |
| MEF2B | MEN1 | MET | MGA | MITF | MLH1 |
| MLH3 | MPL | MRE11A | MSH2 | MSH3 | MSH6 |
| MSI1 | MSI2 | MST1 | MST1R | MTOR | MUTYH |
| MYC | MYCL | MYCN | MYD88 | MYO1B | MYOD1 |
| NAT1 | NAT2 | NBN | NCOA3 | NCOR1 | NEGR1 |
| NF1 | NF2 | NFE2L2 | NFKBIA | NKX2-1 | NKX3-1 |
| NOTCH1 | NOTCH2 | NOTCH3 | NOTCH4 | NPM1 | NRAS |
| NRG1 | NSD1 | NTHL1 | NTRK1 | NTRK2 | NTRK3 |
| NUF2 | NUP93 | OPRM1 | PAK1 | PAK3 | PAK5 |
| PALB2 | PARK2 | PARP1 | PARP2 | PARP3 | PAX5 |
| PBRM1 | PDCD1 | PDCD1LG2 | PDGFRA | PDGFRB | PDK1 |
| PDPK1 | PEG3 | PGR | PHOX2B | PIK3C2B | PIK3C2G |
| PIK3C3 | PIK3CA | PIK3CB | PIK3CD | PIK3CG | PIK3R1 |
| PIK3R2 | PIK3R3 | PIM1 | PLCG2 | PLK1 | PLK2 |
| PMAIP1 | PMS1 | PMS2 | PNRC1 | POLD1 | POLE |
| PPARG | PPM1D | PPP2R1A | PPP2R2A | PPP4R2 | PPP6C |
| PRDM1 | PRDM14 | PREX2 | PRKAR1A | PRKCE | PRKCG |
| PRKCI | PRKD1 | PRKDC | PRRT2 | PRSS8 | PTCH1 |
| PTCH2 | PTEN | PTK2 | PTP4A1 | PTPN11 | PTPRD |
| PTPRS | PTPRT | RAB35 | RAC1 | RAC2 | RAD21 |
| RAD50 | RAD51 | RAD51B | RAD51C | RAD51D | RAD52 |
| RAD54L | RAF1 | RARA | RARB | RASA1 | RASSF1 |
| RASSF8 | RB1 | RBM10 | RECQL | RECQL4 | REL |
| RET | RHBDF2 | RHEB | RHOA | RICTOR | RIT1 |
| RNF43 | ROCK1 | ROS1 | RPS6KA1 | RPS6KA4 | RPS6KB1 |
| RPS6KB2 | RPTOR | RRAGC | RRAS | RRAS2 | RTEL1 |
| RUNX1 | RUNX1T1 | RUNX2 | RXRA | RYBP | SDHA |
| SDHAF2 | SDHB | SDHC | SDHD | SERPINB3 | SERPINB4 |
| SESN1 | SESN2 | SESN3 | SETD2 | SF3B1 | SH2B3 |
| SH2D1A | SHH | SHOC2 | SHQ1 | SLC16A7 | SLIT2 |
| SLX4 | SMAD2 | SMAD3 | SMAD4 | SMARCA1 | SMARCA4 |
| SMARCB1 | SMARCD1 | SMO | SMYD3 | SNAI1 | SNAI2 |
| SNCAIP | SOCS1 | SOS1 | SOX10 | SOX17 | SOX2 |
| SOX9 | SPEN | SPINK1 | SPOP | SPRED1 | SPTA1 |
| SRC | SRSF2 | STAG2 | STAT3 | STAT4 | STAT5A |
| STAT5B | STK11 | STK19 | STK40 | SUFU | SUZ12 |
| SYK | TAF1 | TAP1 | TAP2 | TBX3 | TCEB1 |
| TCF3 | TCF7L2 | TEK | TERT | TET1 | TET2 |
| TFE3 | TFEB | TGFBR1 | TGFBR2 | TMEM127 | TMPRSS2 |
| TNF | TNFAIP3 | TNFRSF14 | TNFSF11 | TOP1 | TOP2A |
| TP53 | TP53BP1 | TP63 | TPX2 | TRAF2 | TRAF7 |
| TSC1 | TSC2 | TSHR | TUBB | TWIST1 | U2AF1 |
| UPF1 | VEGFA | VEGFB | VHL | WEE1 | WHSC1 |
| WHSC1L1 | WISP3 | WNT1 | WNT5A | WNT6 | WRN |
| WT1 | WWTR1 | XIAP | XPA | XPO1 | XRCC2 |
| YAP1 | YES1 | ZBTB2 | ZFHX3 | ZNF217 | ZNF703 |
