# Supplementary material for: Real-world data of pyrotinib-based therapy for patients with brain metastases of HER2-positive advanced breast cancer: a single-center retrospective analysis and molecular portraits
Source: Front Oncol. 2023 Jun 16;13:1105474. doi: 10.3389/fonc.2023.1105474 (PMC10313114; doi:10.3389/fonc.2023.1105474)
Supplement: Supplementary file 6 [file Table_3.docx]

**Table S3. Clinicopathological characteristics of patients and samples.**

| **Patient ID** | **Group** | **Age at diagnosis** | **ER status** | **PR status** | **Metastatic sites (exclude brain metastases)** | **Time before BM occurrence (months)** | **Availability of primary tissue** | **No. plasma samples** |
| --- | --- | --- | --- | --- | --- | --- | --- | --- |
| **P3** | BM | 59 | 1 | 1 | Lung, Bone | 39 | 1 | 2 |
| **P7** | BM | 26 | 0 | 0 | Lung, LN | 28.5 | 1 | 3 |
| **P12** | BM | 33 | 1 | 1 | Liver, Lung, Bone, LN | 31.3 | 0 | 3 |
| **P15** | BM | 31 | 1 | 1 | Lung | 21 | 1 | 2 |
| **P17** | BM | 50 | 0 | 0 | Lung, Bone | 45.3 | 1 | 2 |
| **P18** | BM | 30 | 1 | 0 | Liver, Bone | 12 | 0 | 3 |
| **P19** | BM | 41 | 1 | 0 | Bone | 72 | 0 | 3 |
| **P36** | Non-BM | 45 | 1 | 1 | Liver, Lung, LN | - | 1 | 2 |
| **P37** | Non-BM | 65 | 0 | 0 | Chest wall | - | 0 | 1 |
| **P38** | Non-BM | 45 | 0 | 0 | Liver, Lung, Contralateral breast, LN, Skin | - | 1 | 3 |
| **P39** | Non-BM | 31 | 0 | 0 | Liver, Lung, LN | - | 0 | 2 |
| **P40** | Non-BM | 49 | 0 | 0 | Lung | - | 1 | 2 |
| **P41** | Non-BM | 50 | 0 | 0 | LN | - | 0 | 2 |
| **P42** | Non-BM | 37 | 1 | 1 | Bone, LN | - | 1 | 3 |
| **P43** | Non-BM | 39 | 1 | 1 | Liver, Bone | - | 1 | 1 |
| **P44** | Non-BM | 48 | 0 | 0 | Liver, Bone, LN | - | 0 | 2 |
| **P45** | Non-BM | 32 | 0 | 0 | Lung, LN, Chest wall | - | 0 | 3 |

BM = brain metastases; LN = lymph node.
