# Supplementary material for: Real-world data of pyrotinib-based therapy for patients with brain metastases of HER2-positive advanced breast cancer: a single-center retrospective analysis and molecular portraits
Source: Front Oncol. 2023 Jun 16;13:1105474. doi: 10.3389/fonc.2023.1105474 (PMC10313114; doi:10.3389/fonc.2023.1105474)
Supplement: Supplementary file 7 [file Table_4.docx]

**Table S4. Mutational genes of brain metastases in patients with HER2-positive breast cancer in MSKCC database.**

| **Gene** | **No. samples with one or more mutations** | | **No. profiled samples** | **Frequency (%)** | **Is cancer gene (source: OncoKB)** |
| --- | --- | --- | --- | --- | --- |
| TP53 | | 9 | 12 | 75.00% | Yes |
| KMT2D | | 3 | 12 | 25.00% | Yes |
| H3C6 | | 1 | 5 | 20.00% | Yes |
| MGA | | 1 | 5 | 20.00% | Yes |
| SH2B3 | | 1 | 5 | 20.00% | Yes |
| ANKRD11 | | 1 | 5 | 20.00% | Yes |
| EIF4A2 | | 1 | 5 | 20.00% | Yes |
| EPHA7 | | 1 | 5 | 20.00% | Yes |
| GATA3 | | 2 | 12 | 16.70% | Yes |
| ERBB3 | | 2 | 12 | 16.70% | Yes |
| PIK3CA | | 2 | 12 | 16.70% | Yes |
| TSC2 | | 2 | 12 | 16.70% | Yes |
| PTPRD | | 2 | 12 | 16.70% | Yes |
| ROS1 | | 1 | 12 | 8.30% | Yes |
| PTPN11 | | 1 | 12 | 8.30% | Yes |
| STK11 | | 1 | 12 | 8.30% | Yes |
| RECQL4 | | 1 | 12 | 8.30% | Yes |
| EP300 | | 1 | 12 | 8.30% | Yes |
| NF1 | | 1 | 12 | 8.30% | Yes |
| CDH1 | | 1 | 12 | 8.30% | Yes |
| PRDM1 | | 1 | 12 | 8.30% | Yes |
| GRIN2A | | 1 | 12 | 8.30% | Yes |
| TBX3 | | 1 | 12 | 8.30% | Yes |
| CD276 | | 1 | 12 | 8.30% | Yes |
| LATS1 | | 1 | 12 | 8.30% | Yes |
| PIK3R2 | | 1 | 12 | 8.30% | Yes |
| SOX9 | | 1 | 12 | 8.30% | Yes |
| PBRM1 | | 1 | 12 | 8.30% | Yes |
| EPHA5 | | 1 | 12 | 8.30% | Yes |
| FGFR4 | | 1 | 12 | 8.30% | Yes |
| FGFR2 | | 1 | 12 | 8.30% | Yes |
| FBXW7 | | 1 | 12 | 8.30% | Yes |
| RHOA | | 1 | 12 | 8.30% | Yes |
| APC | | 1 | 12 | 8.30% | Yes |
| FOXA1 | | 1 | 12 | 8.30% | Yes |
| NOTCH1 | | 1 | 12 | 8.30% | Yes |
| SOX2 | | 1 | 12 | 8.30% | Yes |
| MYCN | | 1 | 12 | 8.30% | Yes |
| PTPRS | | 1 | 12 | 8.30% | Yes |

**MSKCC database: Memorial Sloan Kettering Cancer Center, by using the cBio Cancer Genomics Portal (**[**http://cbioportal.org**](http://cbioportal.org)**)**

**OncoKB:** <http://oncokb.org>.
